# HSP70 inhibitors upregulate prostaglandin E_1_-induced synthesis of interleukin-6 in osteoblasts

**DOI:** 10.1371/journal.pone.0279134

**Published:** 2022-12-15

**Authors:** Gen Kuroyanagi, Junko Tachi, Kazuhiko Fujita, Tetsu Kawabata, Go Sakai, Daiki Nakashima, Woo Kim, Kumiko Tanabe, Rie Matsushima-Nishiwaki, Takanobu Otsuka, Hiroki Iida, Osamu Kozawa, Haruhiko Tokuda

**Affiliations:** 1 Department of Orthopedic Surgery, Nagoya City University Graduate School of Medical Science, Nagoya, Japan; 2 Department of Rehabilitation Medicine, Nagoya City University Graduate School of Medical Science, Nagoya, Japan; 3 Department of Pharmacology, Gifu University Graduate School of Medicine, Gifu, Japan; 4 Department of Anesthesiology and Pain Medicine, Gifu University Graduate School of Medicine, Gifu, Japan; 5 Department of Metabolic Research, Research Institute, National Center for Geriatrics and Gerontology, Obu, Aichi, Japan; 6 Department of Clinical Laboratory/Medical Genome Center, National Center for Geriatrics and Gerontology, Obu, Aichi, Japan; Universite de Nantes, FRANCE

## Abstract

Interleukin-6 (IL-6) is a pro-inflammatory and bone-resorptive cytokine that also regulates bone formation. We previously showed that prostaglandin E_1_ (PGE_1_) induces the synthesis of IL-6 by activating p44/p42 mitogen-activated protein kinase (MAPK), stress-activated protein kinase/c-Jun N-terminal kinase (SAPK/JNK), and p38 MAPK in osteoblast-like MC3T3-E1 cells. In the present study, we investigated whether heat shock protein 70 (HSP70), a molecular chaperone that coordinates protein folding and homeostasis, affects PGE_1_-stimulated IL-6 synthesis in MC3T3-E1 cells through the MAPK activation. The osteoblast-like MC3T3-E1 cells were treated with HSP70 inhibitors—VER-155008 and YM-08—, PD98059, SB203580 or SP600125 and then stimulated with PGE_1_. IL-6 synthesis was evaluated using an IL-6 enzyme-linked immunosorbent assay kit. IL-6 mRNA expression was measured by real-time RT-PCR. The phosphorylation of p38 MAPK was evaluated by Western blotting. We found that VER-155008, an HSP70 inhibitor, enhanced the PGE_1_-stimulated IL-6 release and IL-6 mRNA expression. YM-08, another HSP70 inhibitor, also enhanced PGE_1_-stimulated IL-6 release. PD98059, a p44/p42 MAPK inhibitor, and SP600125, a SAPK/JNK inhibitor, upregulated PGE_1_-stimulated IL-6 release. On the other hand, SB203580, a p38 MAPK inhibitor, suppressed PGE_1_-stimulated IL-6 release. YM-08 stimulated the PGE_1_-induced phosphorylation of p38 MAPK. SB203580 suppressed the amplification by YM-08 of the PGE_1_-stimulated IL-6 release. Our results suggest that HSP70 inhibitors upregulate the PGE_1_-stimulated IL-6 synthesis through p38 MAPK in osteoblasts and therefore affect bone remodeling.

## Introduction

Biological stresses such as heat, hypoxia, and extracellular stresses prompt cells to produce heat shock proteins (HSPs), whose cytoprotective functions protect unfolded proteins from aggregating [1]. These molecular chaperones are divided into six groups based on their molecular weight: HSP27, HSP40, HSP60, HSP70, HSP90, and HSP110 [2]. Among them, HSP70 is constitutively expressed in unstressed cells and acts as an ATP-dependent molecular chaperone [3]. HSP70 also contributes to the translocation of the synthesized proteins into organelles across their membranes [4]. HSP70 has been implicated in various diseases including neurodegenerative disease and Alzheimer’s disease [5, 6]; it may also become a therapeutic target of cancer because HSP70 expression is elevated in tissue from the patients with hepatocellular carcinoma and breast cancer [7, 8].

Both osteoblasts and osteoclasts tightly coordinate bone metabolism by forming and resorbing bone, respectively [9]. In the adult skeletal system, old bone is continuously regenerated into new bone by resorption in a process called bone remodeling that maintains the volume and the strength of bone [10]. Disordered bone remodeling, however, causes metabolic bone diseases such as fracture healing distress and osteoporosis. Key members in this process include cytokines, hormones, and growth factors play important roles in bone remodeling [11, 12].

Specifically, interleukin-6 (IL-6) is a multifunctional cytokine that promotes B cell differentiation and T cell activation and induces acute phase proteins [13]. IL-6 not only promotes osteoclastogoenesis and bone resorption indirectly by stimulating receptor activator of nuclear factor κB ligand (RANKL) expression by osteoblasts but also influences bone formation as an osteotropic factor under conditions of increased bone turnover, such as fracture healing [14, 15]. The soluble IL-6 receptor binds IL-6 to promote osteoblast differentiation via the gp130 receptor [16].

Prostaglandin E_1_ (PGE_1_) also regulates bone remodeling [17, 18]. We previously showed that PGE_1_ stimulates the secretion of IL-6 via the cAMP protein kinase A pathway in osteoblast-like MC3T3-E1 cells [19]. PGE_1_ also activates p44/p42 mitogen-activated protein kinase (MAPK), stress-activated protein kinase/c-Jun N-terminal kinase (SAPK/JNK), and p38 MAPK in these cells [20]. We also found that HSP70 inhibitors play various roles in MC3T3-E1 cells: suppressing the migration of MC3T3-E1 cells induced by epidermal growth factor or insulin-like growth factor-I [21, 22] as well as negatively regulating the synthesis of vascular endothelial growth factor (VEGF) in transforming growth factor-β-stimulated MC3T3-E1 cells [23]. However, the role of HSP70 in the PGE_1_-stimulated IL-6 synthesis remains unclear.

In the present study, we investigated how HSP70 inhibitors affect the release of IL-6 in PGE_1_-induced osteoblast-like MC3T3-E1 cells. We found that HSP70 inhibitors upregulated the PGE_1_-stimulated IL-6 synthesis through p38 MAPK in osteoblasts.

## Materials and methods

### Materials

PGE_1_ and the mouse IL-6 enzyme-linked immunosorbent assay (ELISA) kit were purchased from R&D Systems, Inc. (Minneapolis, MN, USA). VER-155008 and YM-08 were obtained from Sigma-Aldrich Co. LLC (St. Louis, MO, USA). PD98059, SB203580, and SP600125 were obtained from Calbiochem-Novabiochem Co. (La Jolla, CA, USA). Phospho-specific p38 MAPK antibodies, p38 MAPK antibodies, and actin were purchased from Cell Signaling Technology, Inc. (Danvers, MA, USA). The enhanced chemiluminescence (ECL) Western blotting detection system was purchased from GE Healthcare Life Sciences (Chalfont, UK). Acrylamide monomer, Tris (hydroxymethyl) aminomethane, sodium dodecyl sulfate (SDS), dithiothreitol, and glycerol were obtained from Nacalai Tesque, Inc. (Kyoto, Japan). Other materials and chemicals were obtained from commercial sources. VER-155008, YM-08, PD98059, SB203580, and SP600125 were dissolved in dimethyl sulfoxide. The maximum concentration of dimethyl sulfoxide was 0.1%, which did not affect the IL-6 assay or protein level detection via Western blotting [19, 23, 24].

### Cell culture

Cloned osteoblast-like MC3T3-E1 cells were established from neonatal mouse calvaria and maintained as previously described [25]. Briefly, the cells were cultured in 10% fetal bovine serum (FBS)-containing α-minimum essential medium (α-MEM) at 37°C in humidified atmospheric air with 5% CO_2_. The cultured cells were seeded on 90-mm diameter dishes (2 × 10^5^ cells/dish) in α-MEM containing 10% FBS. The medium was replaced with α-MEM containing 0.3% FBS at 5 days post-seeding. These cells were used for experiments after 48 h.

### Assay for IL-6

The cultured MC3T3-E1 cells were pretreated with various doses of VER-155008, 10 μM of YM-08, 50 μM of PD98059, 30 μM of SB203580, and 10 μM of SP600125 for 60 min [23, 24], and then stimulated with 10 μM of PGE_1_ in 1 ml of α-MEM containing 0.3% FBS for the indicated periods. The conditioned medium was collected at the end of incubation, and the IL-6 concentration was measured using a mouse IL-6 ELISA kit according to the manufacturer’s instructions [19].

### Real-time RT-PCR

The cultured MC3T3-E1 cells were pretreated with 30 μM of VER-155008 or vehicle for 60 min and then stimulated with 10 μM of PGE_1_ or vehicle in α-MEM containing 0.3% FBS for 2 h [19, 23]. Trizol Reagent (Invitrogen; Thermo Fisher Scientific, Inc. Heysham, Lancashire, UK) and Omniscript Reverse Transcriptase kit (Qiagen Inc., Valencia, CA, USA) were respectively used to isolate total RNA and transcribe it into complementary DNA. Real-time RT-PCR was performed using a LightCycler system (version 3.5; Roche Diagnostics, Basel, Switzerland) in capillaries and Fast Start DNA Master SYBR Green I provided with the kit (Roche Diagnostics). Sense and antisense primers for mouse IL-6 mRNA (primer set ID: MA039013) and GAPDH mRNA (primer set ID: RA015380) were purchased from Takara Bio Inc. (Tokyo, Japan). The amplified products were determined using a melting curve analysis. The IL-6 mRNA level of IL-6 was normalized to that of GAPDH [24]. We have already reported using ELISA that 30 μM of VER-155008 significantly amplified the TGF-β-stimulated VEGF release in osteoblast-like MC3T3-E1 cells [23]. We also demonstrated using real-time RT-PCR that 10 μM of VER-155008 increased the TGF-β-stimulated VEGF mRNA expression levels in the same cells [23]. Therefore, we adopted the same VER-155008 concentrations in this study as described in the aforementioned study for the real-time RT-PCR experiments.

### Western blot analysis

The cultured cells were pretreated with various doses of YM-08 for 60 min and then stimulated with 10 μM of PGE_1_ or vehicle in 1 ml of α-MEM containing 0.3% FBS for 20 min. The cells were then lysed, homogenized, and sonicated in a lysis buffer containing 62.5 mM Tris/HCl, pH 6.8, 2% SDS, 50 mM dithiothreitol, and 10% glycerol. SDS-polyacrylamide gel electrophoresis (PAGE) was performed using the Laemmli method in 10% polyacrylamide gels [26]. The protein was fractionated and transferred onto an Immun-Blot polyvinylidine difluoride membrane (Bio-Rad, Hercules, CA, USA). The membrane was blocked with 5% fat-free dry milk in Tris-buffered saline-Tween (TBS-T; 20 mM Tris/HCl, pH 7.6, 137 mM NaCl, 0.1% Tween 20) for 1 h before incubation with primary antibodies. A Western blot analysis was performed as described previously [27] using antibodies against phospho-specific p38 MAPK, p38 MAPK, and actin as primary antibodies with peroxidase-labeled antibodies raised in goat against rabbit IgG (KPL, Inc., Gaitherburg, MD, USA) being used as secondary antibodies. The primary and secondary antibodies were diluted in TBS-T with 5% fat-free dry milk to optimal concentrations. An X-ray film using an electrochemiluminescence Western blotting detection system was used to visualize peroxidase activity on the membrane; each protein was detected on different gels.

### Densitometric analysis

A densitometric analysis of the Western blots was performed with a scanner and image analysis software program (ImageJ version 1.49, NIH, Bethesda, MD, USA). The phosphorylated levels were calculated as follows: the background-subtracted signal intensity of each phosphorylation signal was normalized to the respective intensity of total protein and plotted as the fold increase relative to that of the control cells without stimulation.

### Statistical analysis

The data were analyzed by an analysis of variance followed by Bonferroni method for multiple comparisons between pairs, and p < 0.05 indicated statistical significant. All data are presented as the mean ± standard error of the mean (SEM) of triplicate determinations from three independent cell preparations.

## Results

### Effect of VER-155008 on the PGE_1_-stimulated IL-6 release in MC3T3-E1 cells

**Fig 1:**
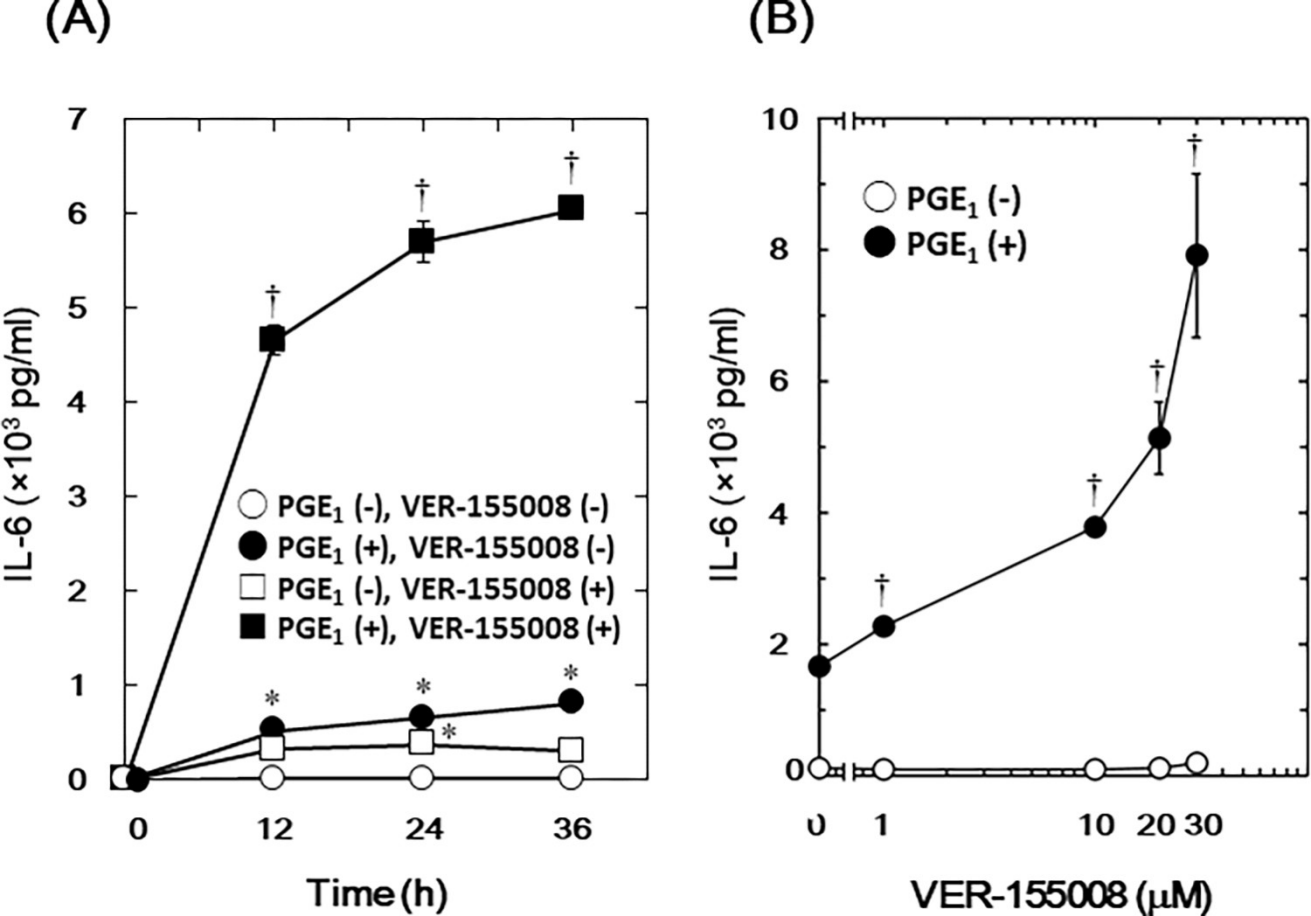
Effect of VER-155008 on the PGE_1_-stimulated IL-6 release in MC3T3-E1 cells. (A) Cultured cells were pretreated with 30 μM of VER-155008 (□,■) or vehicle (○,●) for 60 min and then stimulated with 10 μM of PGE_1_ (●, ■) or vehicle (○, □) for the indicated periods. (B) Cultured cells were pretreated with various doses of VER-155008 for 60 min and then stimulated with 10 μM of PGE_1_ (●) or vehicle (○) for 48 h. IL-6 concentrations of the culture medium were determined by ELISA. Each value represents the mean ± SEM of triplicate determinations from three independent cell preparations. *p < 0.05, compared to the value of control. ^†^p < 0.05, compared to the value of PGE_1_ alone.

We investigated how VER-155008, an inhibitor of HSP70 [28], affected the PGE_1_-stimulated IL-6 synthesis in osteoblast-like MC3T3-E1 cells. We found that VER-155008 significantly enhanced PGE_1_-stimulated IL-6 release (Fig 1A). This amplification was time-dependent up to 36 h, which showed a 500-fold increase in the PGE_1_-stimulated effect. In addition, considerable statistical significance was observed between the group of 30 μM VER-155008 alone (□) and the control group (○) in the IL-6 release at 24 h (Fig 1A). We also found that this enhancement of PGE_1_-stimulated IL-6 release was dose-dependent between 1 and 30 μM in these cells (Fig 1B); VER-155008 at 30 μM elicited an approximately 300-fold increase in the PGE_1_-stimulated IL-6 release.

### Effect of VER-155008 on the PGE_1_-induced expression levels of IL-6 mRNA in MC3T3-E1 cells

**Fig 2:**
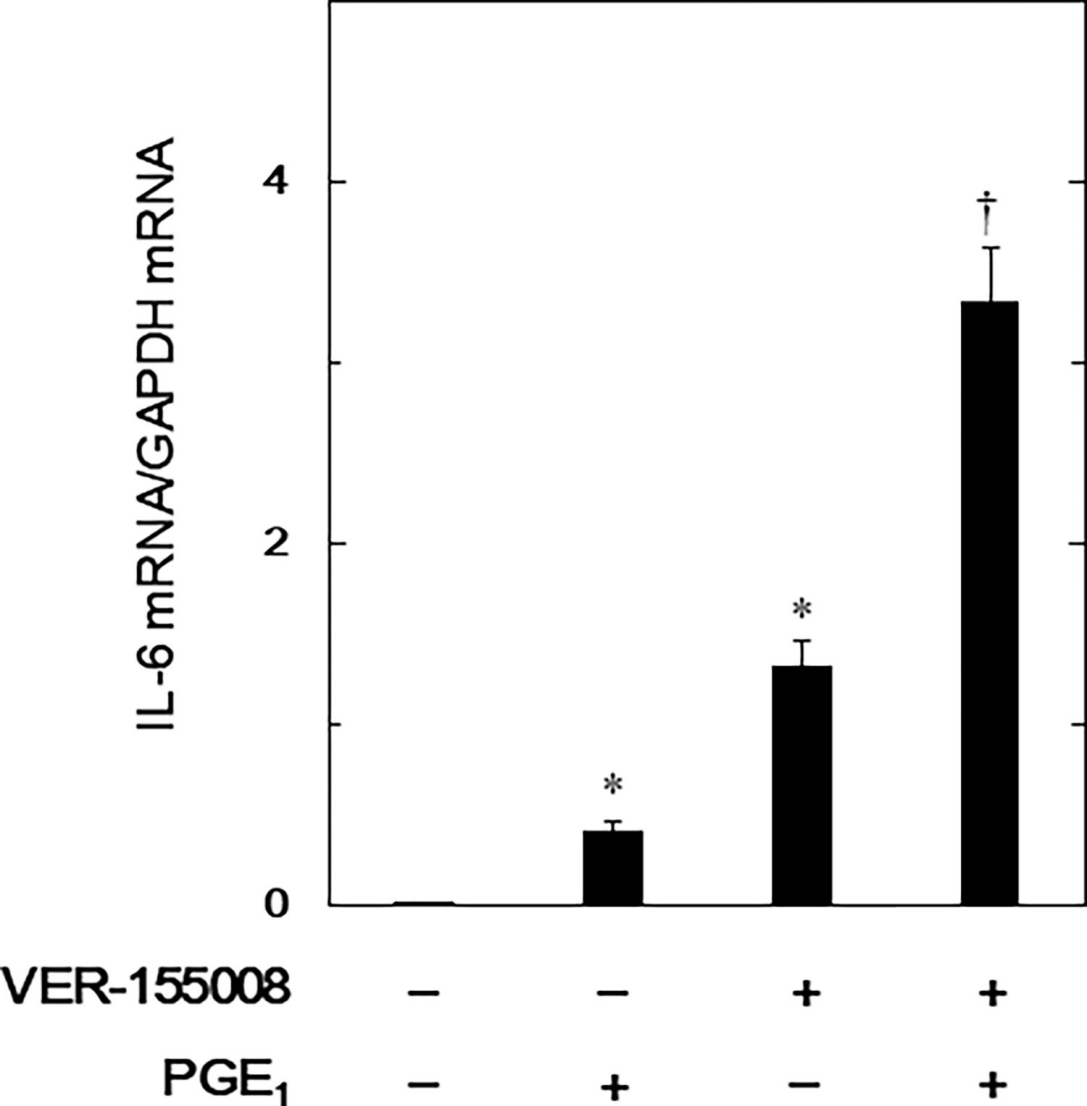
Effect of VER-155008 on the PGE_1_-induced expression levels of IL-6 mRNA in MC3T3-E1 cells. The cultured cells were pretreated with 10 μM of VER-155008 for 60 min and then stimulated with 10 μM of PGE_1_ or vehicle for 2 h. The respective total RNA was then isolated and quantified by real-time RT-PCR. Each IL-6 mRNA value was divided by that of GAPDH mRNA (Vehicle: 0.007 ± 0.001; PGE_1_: 0.412 ± 0.047; VER-155008: 1.317 ± 0.138; PGE_1_+VER-155008: 3.339 ± 0.287). Each value represents the mean ± SEM of triplicate determinations from three independent cell preparations. *p < 0.05, compared to the value of control. ^†^p < 0.05, compared to the value of PGE_1_ alone.

We next quantified PGE_1_-induced mRNA expression of IL-6 with RT-PCR to determine if transcription mediated VER-155008’s amplification of PGE_1_-stimulated IL-6 release. VER-155008 enhanced mRNA expression levels of PGE_1_-induced IL-6 155008 (Fig 2). Interestingly, VER-155008 alone stimulated IL-6 mRNA expression levels although VER-155008 alone did not affect the IL-6 release.

### Effect of YM-08 on the PGE_1_-stimulated IL-6 release in MC3T3-E1 cells

**Fig 3:**
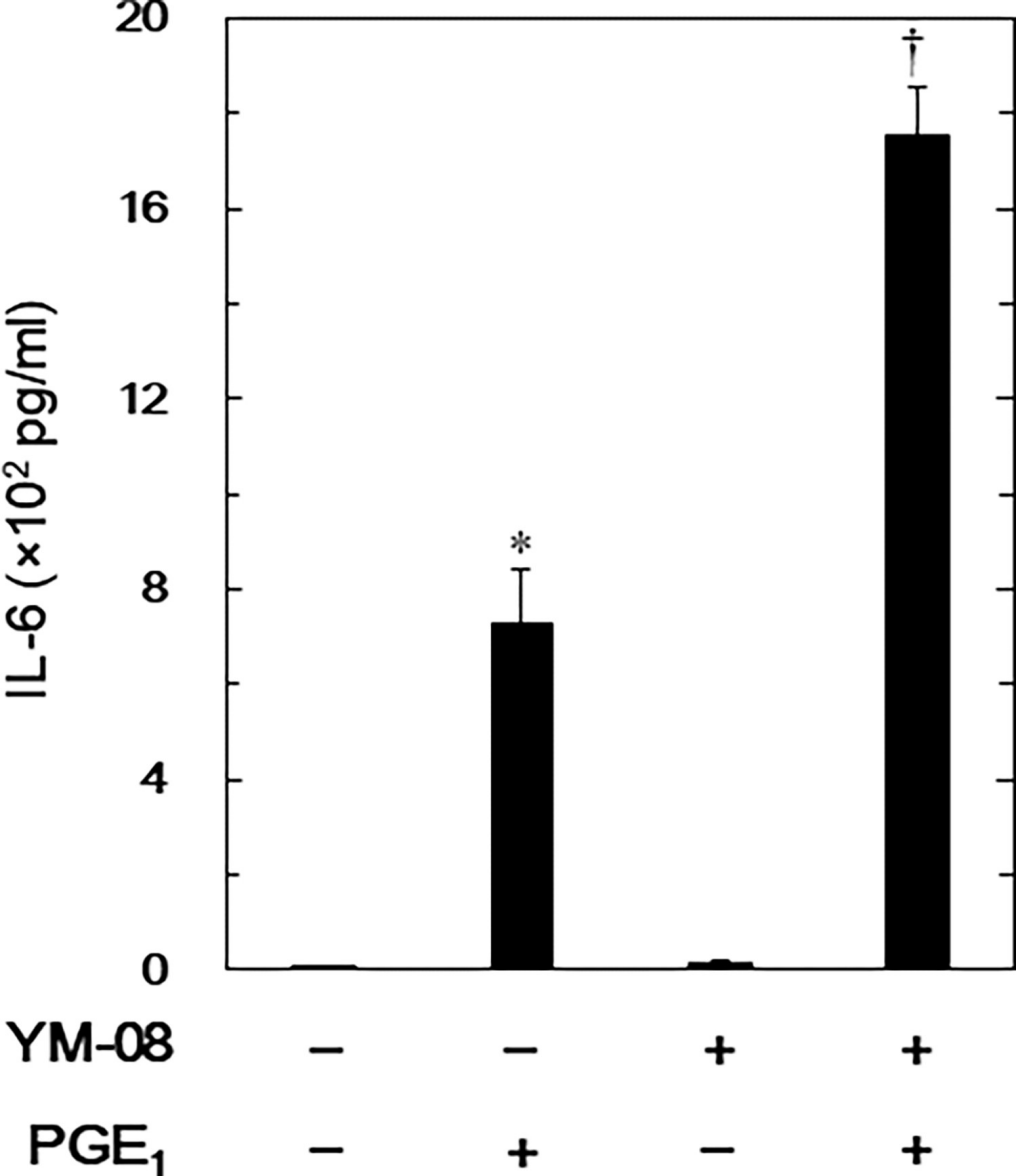
Effect of YM-08 on the PGE_1_-stimulated IL-6 release in MC3T3-E1 cells. The cultured cells were pretreated with 10 μM of YM-08 for 60 min and then stimulated with 10 μM of PGE_1_ or vehicle for 48 h. IL-6 concentrations of the culture medium were determined by ELISA. Each value represents the mean ± SEM of triplicate determinations from three independent cell preparations. *p < 0.05, compared to the value of control. ^†^p < 0.05, compared to the value of PGE_1_ alone.

We studied another HSP70 inhibitor, YM-08 [29], to determine if HSP70 inhibition is indeed the mechanism by which VER-155008 amplifies PGE_1_-stimulated IL-6 release in osteoblast-like MC3T3-E1 cells. Like previous results, YM-08 significantly increased PGE_1_-stimulated IL-6 release in these cells, but YM-08 alone did not affect the release of IL-6 (Fig 3).

### Effects of PD98059, SP600125, or SB203580 on the PGE_1_-stimulated release of IL-6 in MC3T3-E1 cells

We previously showed that PGE_1_ activates the MAPK super family—p44/p42 MAPK, SAPK/JNK, and p38 MAPK—in osteoblast-like MC3T3-E1 cells [20]. We therefore examined whether their respective inhibitors—PD98059, a specific inhibitor of MEK1/2 [30], SP600125, a specific inhibitor of SAPK/JNK [31], and SB203580, a specific inhibitor of p38 MAPK [32]—are involved in PGE_1_-induced IL-6 synthesis. PD98059 or SP600125, which alone hardly affected IL-6 release, significantly enhanced the release of IL-6 stimulated by PGE_1_. However, SB203580, which alone also had little effect on IL-6 release, strongly attenuated the release of IL-6 stimulated by PGE_1_ (Table 1).

**Table 1:** Effects of PD98059, SP600125 or SB203580 on the PGE_1_-stimulated release of IL-6 in MC3T3-E1 cells.

Inhibitor	PGE_1_	IL-6 (pg/ml)
-	-	27 ± 6
-	+	479 ± 37*
PD98059	-	21 ± 1
PD98059	+	808 ± 41^†^
SP600125	-	16 ± 1
SP600125	+	1,521 ± 54^†^
SB203580	-	20 ± 4
SB203580	+	153 ± 9^†^

The cultured cells were pretreated with 50 μM of PD98059, 10 μM of SP600125, or 30 μM of SB203580 for 60 min, then stimulated with 10 μM of PGE_1_ or vehicle for 48 h. The IL-6 concentrations of the culture media were determined using ELISA. Each value represents the mean ± SEM of triplicate determinations from three independent cell preparations.

*p < 0.05, compared to the control.

^†^p < 0.05, compared to PGE_1_ alone.

### Effects of YM-08 on the PGE_1_-stimulated phosphorylation of p38 MAPK in MC3T3-E1 cells

**Fig 4:**
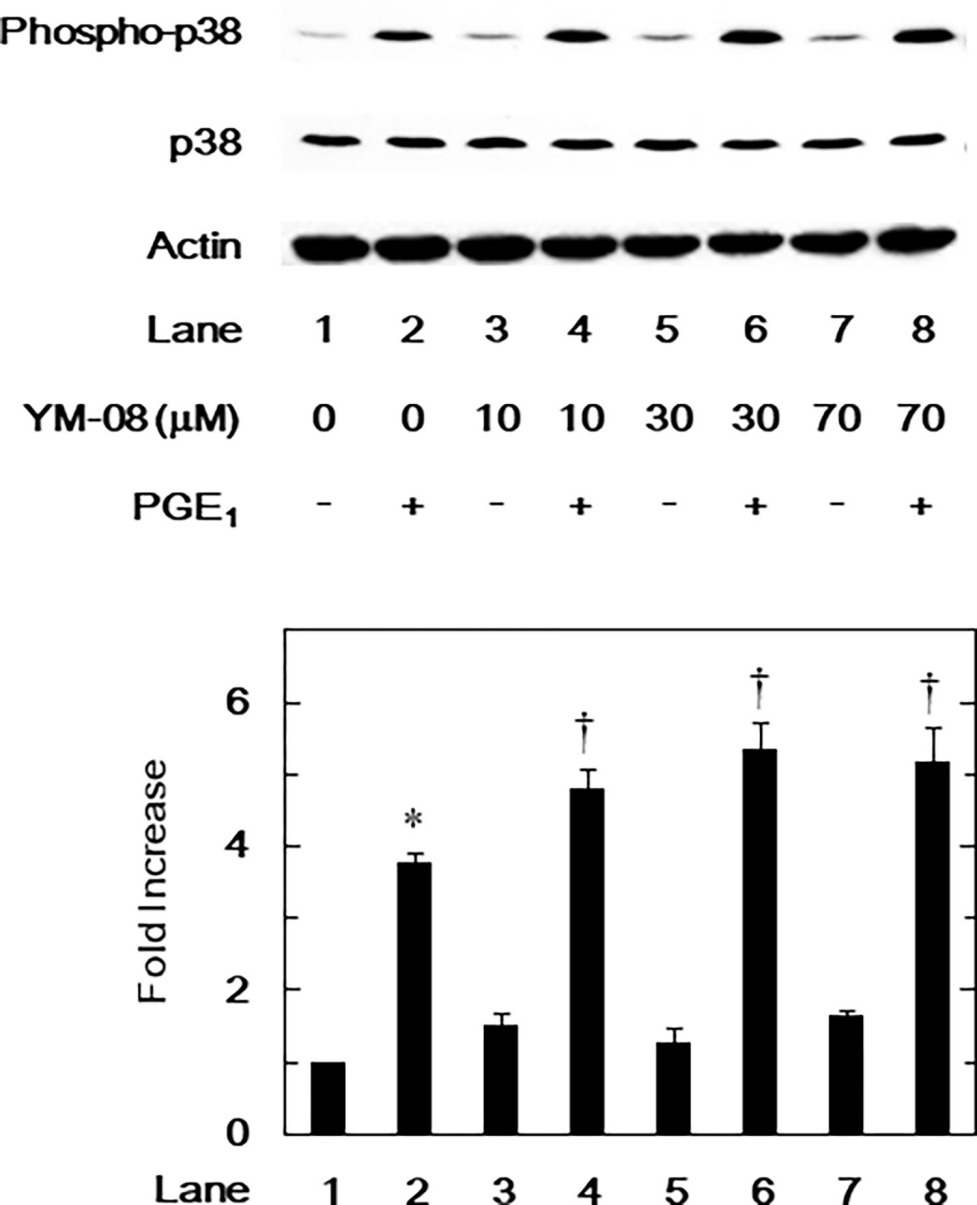
Effects of YM-08 on the PGE_1_-stimulated phosphorylation of p38 MAPK in MC3T3-E1 cells. The cultured cells were pretreated with 10 μM of YM-08 for 60 min and then stimulated with 10 μM of PGE_1_ or vehicle for 20 min. The cell extracts were then subjected to SDS-PAGE and subsequent Western blot analysis with antibodies against phospho-specific p38 MAPK, p38 MAPK, and actin. The histogram quantitatively represents the PGE_1_-induced levels obtained from laser densitometric analysis of three independent experiments. Each value represents the mean ± SEM of triplicate determinations from three independent cell preparations. *p < 0.05, compared to the value of control. ^†^p < 0.05, compared to the value of PGE_1_ alone.

After finding that only SB203580 weakened PGE_1_-induced IL-6 synthesis, we further examined the effect of YM-08 on the PGE_1_-induced phosphorylation of p38 MAPK. Concentrations of YM-08 between 10 and 70 μM significantly enhanced the PGE_1_-induced phosphorylation of p38 MAPK (Fig 4).

### Effects of SB203580 on YM-08’s amplification of PGE_1_-stimulated IL-6 release in MC3T3-E1 cells

**Fig 5:**
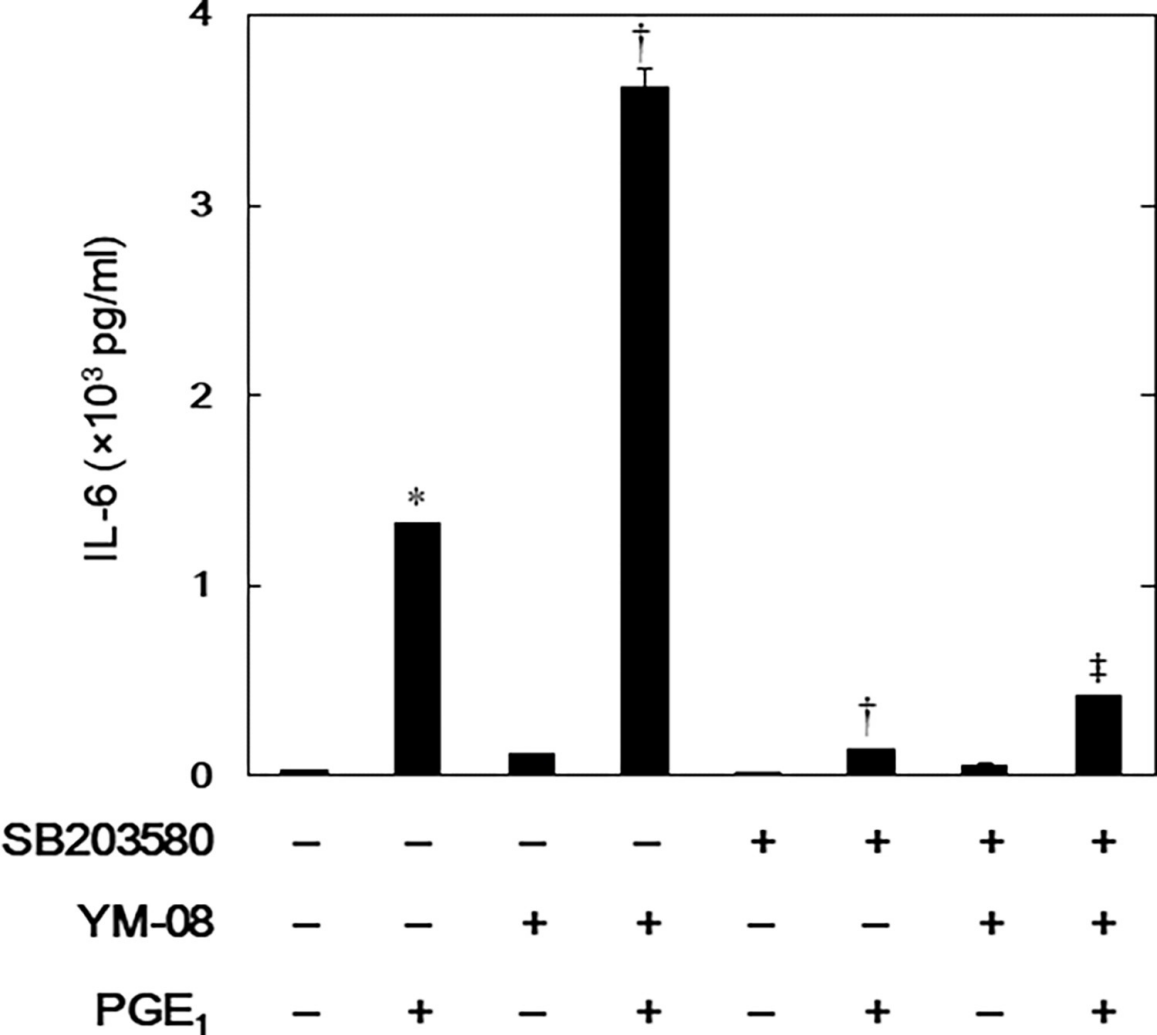
Effects of SB203580 on YM-08’s amplification of PGE_1_-stimulated IL-6 release in MC3T3-E1 cells. The cultured cells were preincubated with 30 μM of SB203580 or vehicle for 60 min, subsequently pretreated with 10 μM of YM-08 or vehicle for 60 min, and then stimulated with 10 μM of PGE_1_ or vehicle for 48 h. IL-6 concentrations of the conditioned media were determined by ELISA. Each value represents the mean ± SEM of triplicate determinations from three independent cell preparations. *p < 0.05, compared to the value of control. ^†^p < 0.05, compared to the value of PGE_1_ alone. ^‡^p < 0.05, compared to the value of PGE_1_ with YM-08 pretreatment.

Finally, we examined how SB203580 affects YM-08’s enhancement of the PGE_1_-stimulated IL-6 release in osteoblast-like MC3T3-E1 cells. SB203580 significantly suppressed YM-08’s amplificatory effect (Fig 5).

## Discussion

Here, we showed that HSP70 inhibitors VER-155008 and YM-08 upregulate PGE_1_-stimulated IL-6 synthesis through p38 MAPK in osteoblasts. PGE_1_ also upregulated the expression levels of IL-6 mRNA, which VER-155008 further enhanced. Our findings suggest that HSP70 inhibitor enhances PGE_1_-stimulated IL-6 release at a point upstream of transcriptional levels in osteoblast-like MC3T3-E1 cells. However, we observed considerable statistical significance between the group of 30 μM VER-155008 alone (□) and the control (○) in the case of IL-6 release at 24 h, which seemed to be consistent in part with the VER-155008 effect on the IL-6 mRNA expression. Regarding the IL-6 mRNA expression levels, VER-155008-mediated upregulation by itself was higher than that influenced by PGE_1_ alone, whereas the PGE_1_-mediated IL-6 release levels were higher than those affected by VER-155008. Concerning the VER-155008 effect on mRNA expression, VER-155008 reportedly enhances toll-like receptor 5 (TLR5) mRNA expression but reduces TLR5 cell surface expression in human myeloid leukemia THP-1 cells, suggesting that suppressing the HSP70 inhibitor-related chaperone function could prevent mature TLR5 traffic from the endoplasmic reticulum (ER) to the cell surface [33]. Therefore, the discrepancy between how VER-155008 and PGE_1_ affect the IL-6 mRNA expression levels and IL-6 release might be caused by the VER-155008-induced suppression of mature IL-6 translocation from the ER to secretory granules by VER-155008 in osteoblasts.

We then explored the mechanism behind PGE_1_-stimulated IL-6 synthesis and the amplificatory effect of HSP70 inhibitors in osteoblast-like MC3T3-E1 cells. PGE_1_ stimulates the activation of three major MAPKs in osteoblast-like MC3T3-E1 cells: p44/p42 MAPK, p38 MAPK, and SAPK/JNK [20]. Additionally, p38 MAPK and SAPK/JNK but not p44/p42 MAPK positively regulate PGE_1_-stimulated VEGF synthesis [20]. In this study, we studied the roles of p38 MAPK, SAPK/JNK and p44/p42 MAPK in the IL-6 synthesis of MC3T3-E1 cells; only SB203580, a p38 MAPK inhibitor, significantly reduced PGE_1_-stimulated IL-6 release, whereas PD98059, a p44/p42 MAPK inhibitor, and SP600125, a SAPK/JNK inhibitor, enhanced the release of IL-6. These results indicate that p38 MAPK functions as a positive regulator, and p44/p42 MAPK and SAPK/JNK function as negative regulators in PGE_1_-stimulated IL-6 synthesis in osteoblast-like MC3T3-E1 cells.

We sought to elucidate the exact mechanism by which HSP70 inhibitor upregulates PGE_1_-stimulated IL-6 synthesis in osteoblast-like MC3T3-E1 cells. We found that YM-08 markedly enhanced the PGE_1_-induced phosphorylation of p38 MAPK. SB203580 also suppressed YM-08’s amplification of PGE_1_-stimulated IL-6 release. Based on these findings, it is likely that the activation of p38 MAPK in osteoblast-like MC3T3-E1 cells mediates this process. Related to the PGE_1_-elicited p38 MAPK pathway in osteoblast-like MC3T3-E1 cells, we have previously reported that cAMP/protein kinase A pathway exists upstream of p38 MAPK and is involved in p38 MAPK activation [34]. The adenylate cyclase activity triggers cAMP formation from ATP, resulting in protein kinase A activation. In this study, HSP70 inhibitor increased the PGE_1_-induced p38 MAPK phosphorylation and upregulated the PGE_1_-stimulated IL-6 synthesis. Taking these findings into account as a whole, it is likely that HSP70 might affect cAMP/protein kinase A pathway and consequently regulate p38 MAPK phosphorylation in osteoblast-like MC3T3-E1 cells. Further investigation would be required to elucidate the possible underlying mechanism of how HSP70 regulates p38 MAPK phosphorylation in osteoblasts. However, only YM-08 but not VER-155008 was used to evaluate PGE_1_-stimulated p38 MAPK phosphorylation in this study. Unfortunately, no data on the use of VER-155008 to confirm the enhancing effects of YM-08 on the PGE_1_-stimulated p38 MAPK phosphorylation is available. Further investigations using VER-155008 for the PGE_1_-stimulated p38 MAPK phosphorylation would be necessary to provide additional support to our findings.

IL-6 is a pro-inflammatory cytokine that governs bone remodeling under physiological and pathological conditions by inducing RANKL expression and therefore osteoclastogenesis as well as promoting bone resorption [14, 15]. A recent study suggests that IL-6 is necessary for bone formation and functions as an osteotropic factor during increased bone turnover [15]. On the other hand, prostaglandin, a bone-resorptive cytokine, also mediates bone remodeling and formation [35]. Bone resorption by osteoclasts initiates bone remodeling, after which osteoblasts form bone. Proper bone remodeling is essential for maintaining bone quality and bone volume. IL-6 is currently known to mediate bone remodeling from the viewpoint of whole bone metabolism, and osteoblast-like MC3T3-E1 cells normally express HSP70 even when not stimulated [36]. Accounting for these findings, our results show that HSP70 functions in not only protein folding and proteostasis but also bone metabolism. HSP70 is implicated in neurodegenerative diseases and cancer; HSP70 activity is a potential drug target [5]. We showed that HSP70 inhibitors enhance PGE_1_-stimulated IL-6 synthesis in osteoblasts and therefore could affect bone metabolism as a modulator of bone remodeling through IL-6 synthesis in osteoblasts.

In conclusion, our results strongly suggest that the HSP70 inhibitors upregulate PGE_1_-stimulated IL-6 synthesis through p38 MAPK in osteoblasts. Further investigations should further explore how HSP70 functions in osteoblasts.

## Supporting information

S1 Raw images Full-length gels and blots of Fig 4. (PDF)
