# Supplementary figures and images for: HSP70 inhibitors upregulate prostaglandin E1-induced synthesis of interleukin-6 in osteoblasts
Source: PLoS One. 2022 Dec 15;17(12):e0279134. doi: 10.1371/journal.pone.0279134 (PMC9754267; doi:10.1371/journal.pone.0279134)

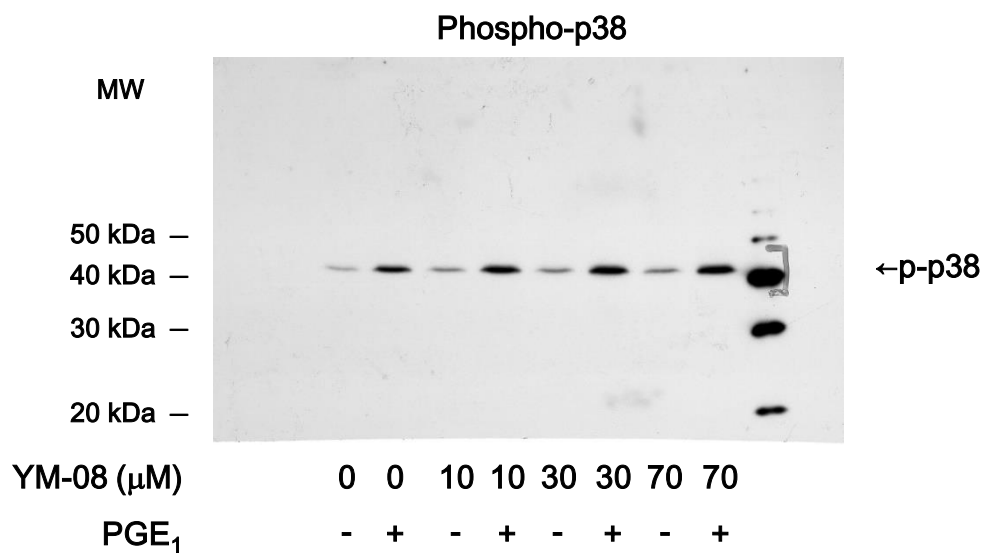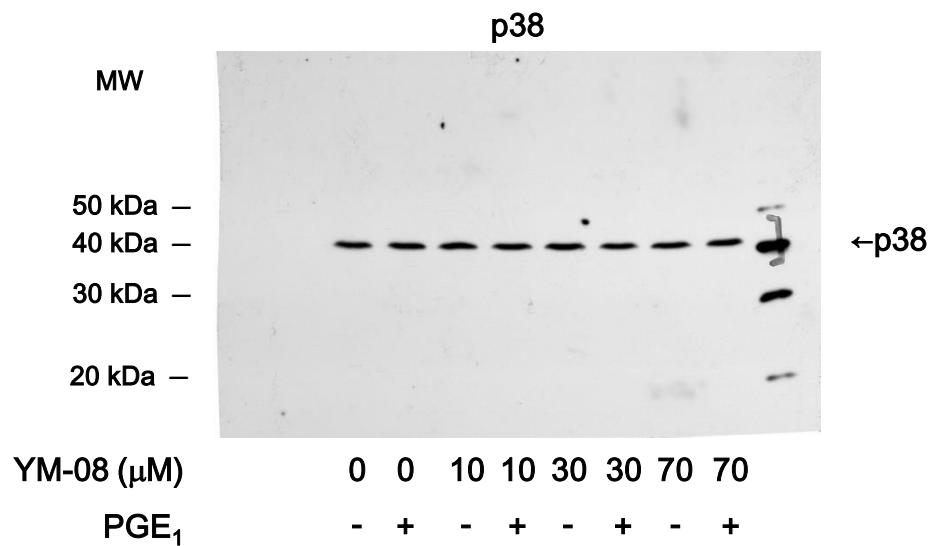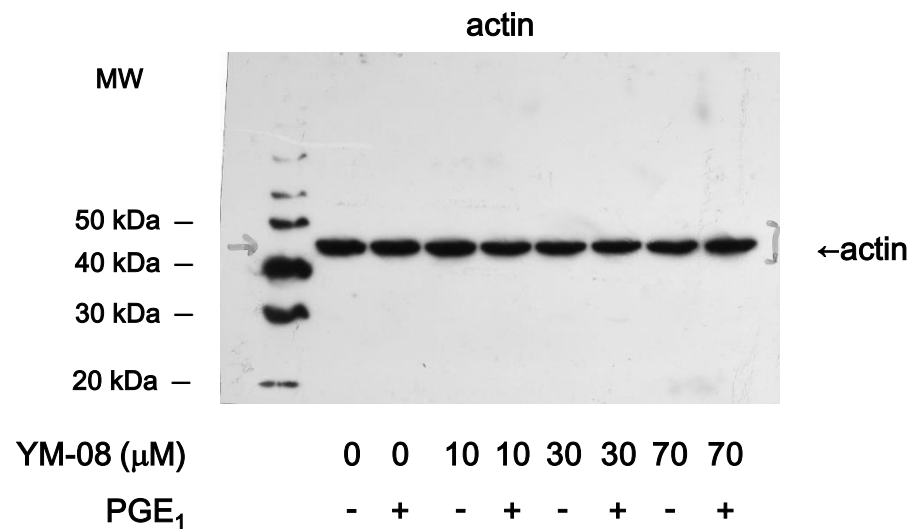

Supplement: S1 Raw images — (PDF) [file pone.0279134.s001.pdf]
